# Explorability and the origin of network sparsity in living systems

**DOI:** 10.1038/s41598-017-12521-1

**Published:** 2017-09-26

**Authors:** Daniel M. Busiello, Samir Suweis, Jorge Hidalgo, Amos Maritan

**Affiliations:** Department of Physics and Astronomy, University of Padova, CNISM and INFN, 35131 Padova, Italy

## Abstract

The increasing volume of ecologically and biologically relevant data has revealed a wide collection of emergent patterns in living systems. Analysing different data sets, ranging from metabolic gene-regulatory to species interaction networks, we find that these networks are sparse, i.e. the percentage of the active interactions scales inversely proportional to the system size. To explain the origin of this puzzling common characteristic, we introduce the new concept of explorability: a measure of the ability of an interacting system to adapt to newly intervening changes. We show that sparsity is an emergent property resulting from optimising both explorability and dynamical robustness, i.e. the capacity of the system to remain stable after perturbations of the underlying dynamics. Networks with higher connectivities lead to an incremental difficulty to find better values for both the explorability and dynamical robustness, associated with the fine-tuning of the newly added interactions. A relevant characteristic of our solution is its scale invariance, i.e., it remains optimal when several communities are assembled together. Connectivity is also a key ingredient in determining ecosystem stability and our proposed solution contributes to solving May’s celebrated complexity-stability paradox.

## Introduction

In inanimate matter, elementary units, such as spins or particles, always have their mutual interactions turned on (with the intensity decaying with their relative distance). Thus, the interaction network is dense, with all connections present, i.e. particles do not have the freedom to adjust or change their interactions unless they change their relative distances. In contrast, living systems are composed of interacting entities, such as genes^1–4^, metabolites^1,5,6^, individuals^7–9^, and species^4,10–14^, with the ability to rearrange and tune their own interactions in order to achieve a desired output^1^. Indeed, thanks to advances in experimental techniques, which are generating an increasing volume of publicly available ecologically and biologically relevant data, several studies indicate that interaction networks in living systems possess a non-random architecture characterised by the emergence of recurrent patterns and regularities^10,11,15,16^.

**Figure 1:**
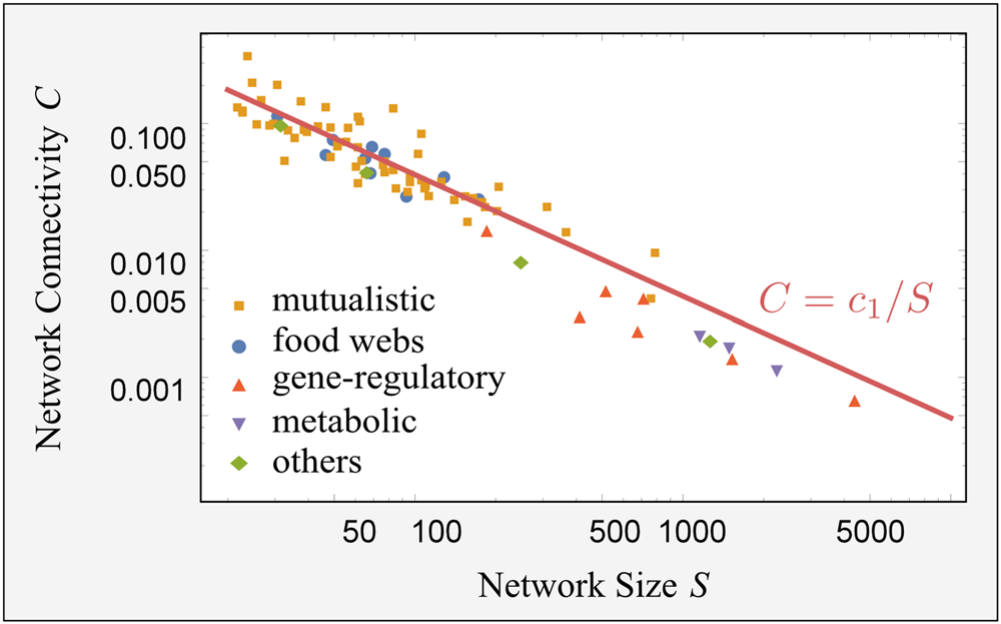
Sparsity of interactions in living systems. The connectivity, *C*, is defined as the fraction of active interactions among the *S* ^2^ possible links (including self-interactions), whereas the system size, *S*, refers to the number of nodes in the graph. We plotted *C* as a function of *S* in log-scale for 83 biological networks (ecological mutualistic communities and food webs, gene-regulatory networks, metabolic networks and others). The data show a clear emergent pattern of sparsity in empirical biological networks, as evidenced by the red line $C={c}_{1}/S$ (with ${c}_{1}=3.8\pm 0.2$). Details on the database are given in the Supplementary Information.

Analysing different data sets of ecological, gene-regulatory, metabolic and other biological interaction networks^1,2,11,12,17–19^, (see Supplementary Information for details), we find that one ubiquitous emergent pattern is sparsity, i.e. the percentage of the active interactions (connectivity) scales inversely proportional to the system size (illustrated in Fig. 1). For example, in the case of ecological systems, species interact selectively even when they coexist at short distances and most of the interactions are turned off. A generic system formed by *S* interacting units may have a maximum number of interactions equal to *S* ^2^ (including self-interactions), i.e. a connectivity *C* (defined as the fraction of active interactions) equal to 1. On the other hand, the minimum number of interactions that guarantees that the interaction network is connected is of the order of *S*, that is $C\sim \mathrm{1/}S$, corresponding to the percolation threshold of random networks^20^. Thus, in this range of possible connectivities, it is quite surprising that the observed ones in the analysed interaction networks all correspond to the lowest possible values. However, it is not known if this recurrent property gives any advantage or reward to the system and a theoretical framework to understand the origin of sparsity is still lacking.

Guided by such an intriguing observation, the main goal of our work is to shed some light on why this pattern emerges and to study, from a theoretical point of view, if the sparsity of interactions confers any advantage to the system. In this context, variational principles have been proven to be a useful tool to elucidate some of the recurrent patterns in nature^11,21^, In the same vein, in this work we propose an optimisation approach to describe the role of active interactions in living systems. We show that sparse networks offer, at the same time, a maximum capability of the system to visit as many stable attractors as possible by simply tuning the interaction strengths (explorability), as well as the largest robustness of the underlying dynamics, guaranteeing that such attractors remain stable (dynamical robustness).

## Results

### Mathematical framework

We consider a system composed of *S* nodes (e.g. species, metabolites, genes) characterised by dynamical variables, ${\bf{x}}=({x}_{1},{x}_{2},\mathrm{...,}\,{x}_{S})$ (e.g. populations, concentrations, levels of expression), following a generalised Lotka-Volterra (GLV) dynamics:

1 $${\dot{x}}_{i}={G}_{i}({x}_{i}){F}_{i}(\sum _{j=1}^{S}{w}_{ij}{x}_{j})\quad {\rm{for}}\,i=\mathrm{1,}\ldots ,S\mathrm{.}$$

In the simplest case ${G}_{i}(x)=x,{F}_{i}(x)={\alpha }_{i}+x$, we recover the classic *S* species Lotka-Volterra equations and we refer to the parameter *α*_*i*_ as the growth rate. In this case the interaction of node *i* with node *j* is encoded in the matrix element ${w}_{ij}$, whose diagonal entries set the scale of the interaction strengths, which for the sake of simplicity^22^ we set to −1. For convenience, we also introduce the adjacency matrix ${A}_{ij}$, whose entries are 1 if the corresponding ${w}_{ij}\ne 0$ and 0 otherwise. A non-trivial stationary point of the dynamics of Eq. (1), ${{\bf{x}}}^{\ast }$, is determined by the interactions within the system, i.e. when ${F}_{i}({\sum }_{j=1}^{S}{w}_{ij}{x}_{j}^{\ast })=0$, ${{\bf{x}}}^{\ast }=-{{\bf{w}}}^{-1}{\boldsymbol{\alpha }}$, and its stability is guaranteed if all the eigenvalues of the Jacobian matrix evaluated at this point, ${J}_{ij}={x}_{i}^{\ast }{w}_{ij}$, have a negative real part. GLV dynamics have been used to model the time evolution of ecological systems^22,23^, human microbiota dynamics^24^, gene expression^25^ and other biological systems^26^, where *x*_*i*_ represents the density of the *i*-th species, and therefore we focus on the stable and feasible stationary solutions of the dynamics^19,22^, (${x}_{i}^{\ast } > 0$). For simplicity, here we focus on the simpler case with ${G}_{i}(x)=x,{F}_{i}(x)={\alpha }_{i}+x$, but in the Supplementary Information we recast all the results for the more general non-linear dynamics, Eq. (1).

### Explorability

Depending on the model parameters, the dynamics of (1) exhibits different fixed points, ${\dot{x}}_{i}^{\ast }=0$, that may correspond to feasible/non-feasible and either stable/unstable solutions. We do not study more complicated possibilities such as limit cycles or chaotic strange attractors, as this would require to go beyond linear stability analysis. Focusing on the interaction strengths as our tuning parameters, by varying them we can pass from one fixed point to another, and change its feasibility and stability. In brief, we take the explorability as the volume of feasible and stable fixed points spanned by modifying the link weights, while all other model parameters are kept fixed. The explorability resembles the concepts of robustness and adaptability in the context of evolutionary dynamics, which make up the number of ‘phenotypes’ (attractors of the dynamics) that can be reached by mutations in the space of ‘genotypes’ (interaction networks)^27,28^, Nevertheless, in our case the system has only one stable and feasible fixed point (‘phenotype’) once the weights *w*_*ij*_ are fixed (*w* is invertible). Therefore, for any change of a link weight (a ‘mutation’ of *w*) we have a different fixed point (‘phenotype’), i.e. in our setting there are not ‘neutral’ mutations.

**Figure 2:**
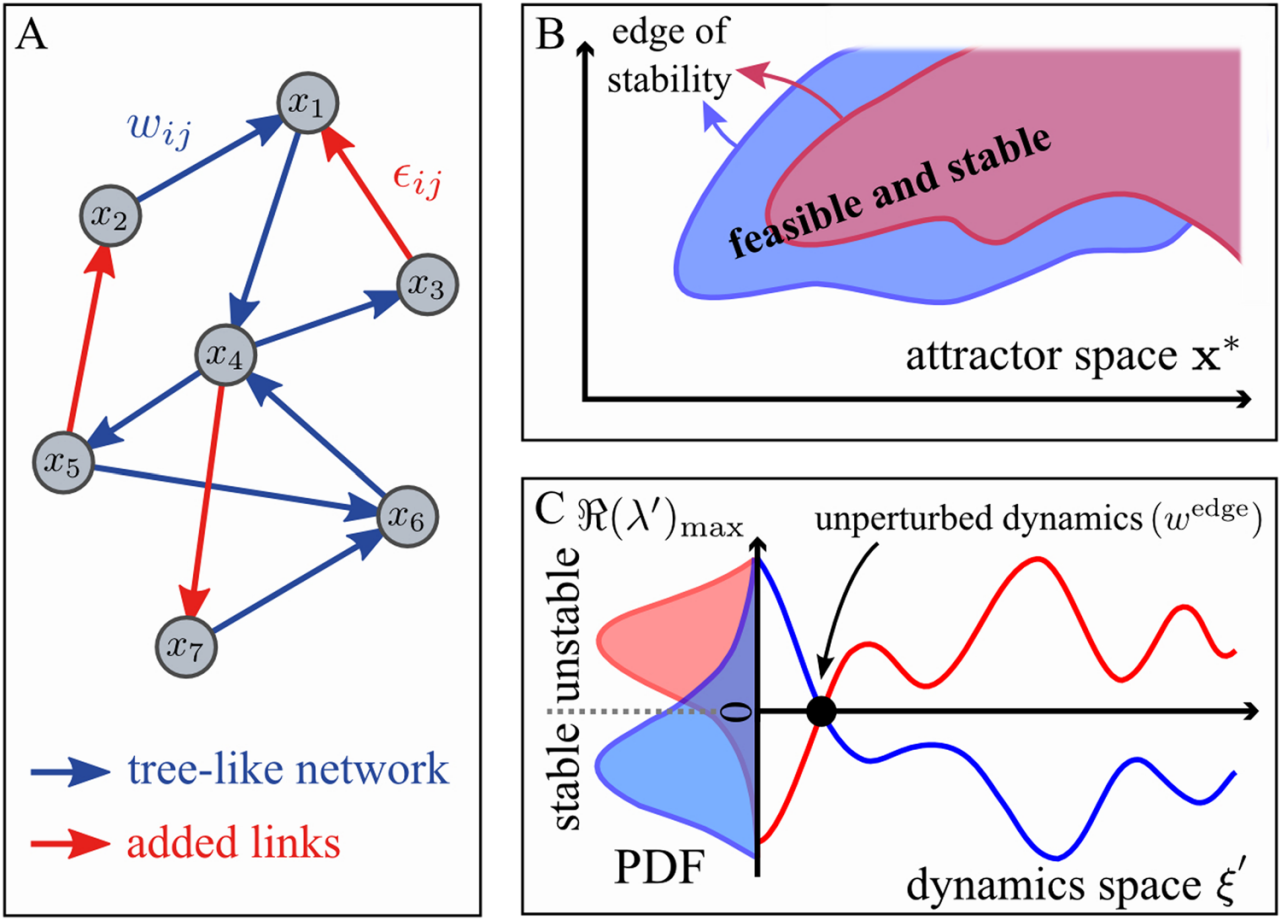
Measuring explorability and dynamical robustness. We considered a system of *S* nodes representing species, genes, metabolites,… (*S* = 7 in the picture), whose state ${\bf{x}}=({x}_{1},\mathrm{...,}\,{x}_{S})$ obeys a non-linear dynamics of the type ${\dot{x}}_{i}={G}_{i}({x}_{i}){F}_{i}({\sum }_{j=1}^{S}{w}_{ij}{x}_{j})$, with *w*_*ij*_ encoding the network structure and the strength of the interactions. (Panel A) We started from a tree-like network, i.e. a tree with one loop, with *S* links, represented by the blue-colored links, and, for such a topology, we searched for the feasible and stable fixed points as we varied the interaction strengths. (Panel B) The spanned volume sketched by the blue-shaded region (a 2D projection of the *S*-dimensional space) corresponds to the network explorability. (Panel C) The dynamical stability quantifies the robustness of the system to perturbations of the dynamics itself, $({\bf{G}}+\delta {\bf{G}})({\bf{F}}+\delta {\bf{F}})$, and should not be confused with the standard stability or resilience that measures the response to perturbations on the fixed point (which is already implicit in the measure of explorability). Starting from an interaction matrix whose fixed point of the underlying dynamics is at the edge of stability, denoted by ${w}_{ij}^{{\rm{edge}}}$ (for which $\Re {(\lambda )}_{\max }=0$, black dot in the picture), the dynamics was perturbed, and the stability of the new fixed point was evaluated in order to test the dynamical robustness of the system. As demonstrated in the Supplementary Information, this can be simply encompassed by computing the principal eigenvalue $\Re {(\lambda ^{\prime} )}_{{\rm{\max }}}$ of the perturbed Jacobian matrix, ${J^{\prime} }_{ij}={\xi ^{\prime} }_{i}{w}_{ij}$, where $\xi ^{\prime}$ depends on the details of the perturbation, that we take to be a random vector. The histogram of $\Re {(\lambda ^{\prime} )}_{{\rm{\max }}}$ is sketched in panel C as the dynamics $\xi ^{\prime}$ is varied. Finally, we increased the connectivity of the network by including additional fixed strengths, ${\varepsilon }_{ij}$ (red edges in the graph), to the network. The same previous analysis was performed again by varying the strengths of the blue links and the corresponding results are shown in red in the panels on the right. By randomly sampling the location and strengths of the added links, we investigate if the explorability and dynamical robustness statistically increase or diminish for higher values of the connectivity.

More specifically, we can define the explorability for interacting systems described by a GLV dynamics Eq. (1) and for a given topology (i.e., an ensemble of interaction networks having the same adjacency matrix *A*, but in general different links weights *w*_*ij*_) of the interaction network. The explorability *V*_*E*_ is the volume in ${{\mathbb{R}}}^{S}$ spanned by all the feasible and stable fixed points as one varies *w*_*ij*_ while keeping all other parameters fixed (see Fig. 2). Notice that, with such a definition, the fully connected network has the largest possible explorability, since any other topology is attainable by making some of the matrix entries arbitrarily close to zero. However, might the optimal or quasi-optimal solutions indeed be the ones where most of the interactions are turned off, as suggested by the observational data? Moreover, in the fully connected case, many interaction parameters have to be specified (there are *S* ^2^ matrix elements that can be varied), and then spanning all the possible fixed points becomes a complicated, fine-tuning problem, which does not seem to be feasible in biological systems^29^. Therefore, we pose the following questions: what is the relationship between explorability and the interaction network topology? Is there an optimal network structure that maximises explorability? To answer these questions, we started by analysing the extreme case of a sparse topology with just *S* links, i.e. a tree with one loop with connectivity *C* = 2/*S* (see Fig. 2) (the factor 2 comes from the fact that we also count the self-interactions). For the sake of simplicity in what follows we will refer to this topology as the *tree-like network*. Even in this simple case, measuring the explorability requires us to scrutinise an *S*-dimensional space of parameters (corresponding to the *S* entries of the interaction matrix). Furthermore, one still has to choose the values of *α*_*i*_, which are intrinsic parameters of the dynamics (e.g. species growth rates) and should be set *a priori* (in contrast to the interactions *w*_*ij*_, which are tuning parameters in our approach). For this reason, we introduced various degrees of approximations in our setting. We first considered the simplest uni-parametric case, ${\alpha }_{i}=\alpha$, that we can fix without a loss of generality to *α* = 1 (we checked that our conclusions are still valid for other choices of *α*_*i*_, see below). In addition, we first restricted the analysis to the subspace of fixed points with homogeneous components, i.e. ${x}_{i}^{\ast }={x}^{\ast }$. Under these approximations, computing the explorability becomes a much simpler task and we were able to develop an analytical solution to this problem (see Methods). This approach, although *a priori* seems too drastic, leads to rather reasonable estimates of the explorability. As a second step, we enlarged the region explored by introducing some heterogeneity into the components of x*.

Most probably, the volume of feasible and stable fixed points may become infinite. However, we are always interested in comparing such volumes for different topologies. Indeed, in the simple homogeneous situation, we observed that in almost all the cases (100 per cent for tree-like networks and, e.g., more than 98 per cent for *C* = 0.5), we can identify two regions along ${x}_{i}^{\ast }={x}^{\ast }$: fixed points become unstable for small x* and stable for large x*, with a marginally stable fixed point intersecting at a single value ${x}_{c}^{\ast }$, for which $\Re {(\lambda )}_{\max }={\max }_{i=\mathrm{1,..,}S}\Re ({\lambda }_{i})\,=\,0$, where ${\lambda }_{i}$ are the eigenvalues of the Jacobian matrix *J* (see Supplementary Information for more details). Therefore, we can take ${V}_{E}={V}_{0}-{x}_{c}^{\ast }$ as a proxy for the explorability, where *V*_0_ is a sufficiently large constant (*V*_0_ = 1 in our analysis), which allows for comparison between different topologies. With this definition, we can compute analytically the explorability of a tree-like network with *S* links (see Supplementary Information), finding that, among all the possible tree-like topologies, the one with just a loop composed of three nodes leads to the optimal explorability, ${V}_{E}=\mathrm{2/3}$. This structure (which we refer to as the optimal tree-like network) constitutes our reference network when increasing the connectivity.

As a third step, we analysed the explorability of networks with higher connectivities. This enormously increases the number of matrix entries that have to be modified when computing the spanned volume of feasible and stable fixed points. For this reason, we adopted the following approach (see Fig. 2): starting from the tree-like topology, we introduced *additional* links to the tree-like topology of weights ${\varepsilon }_{ij}$ for any extra links between nodes *i* and *j*, and then computed the explorability, fixing the values of all ${\varepsilon }_{ij}$ and tuning the other matrix elements. Sampling different values of the added link weights (but not their locations), we can construct a histogram of the explorabilities, $P({V}_{E}|\{{\varepsilon }_{ij}\})$. In addition, we are not interested in distinguishing different topologies with the same connectivity *C*, so we also sampled over different locations of the added links, leading to $P({V}_{E}|C)$ (see Methods for technical details). For numerical reasons, we have tested that our results are robust for network sizes *S* < 100. However, it is important to mention that network size is a significant factor in the spectrum of fixed points^30^ and that more complex phenomenology could be found in very large systems.

**Figure 3:**
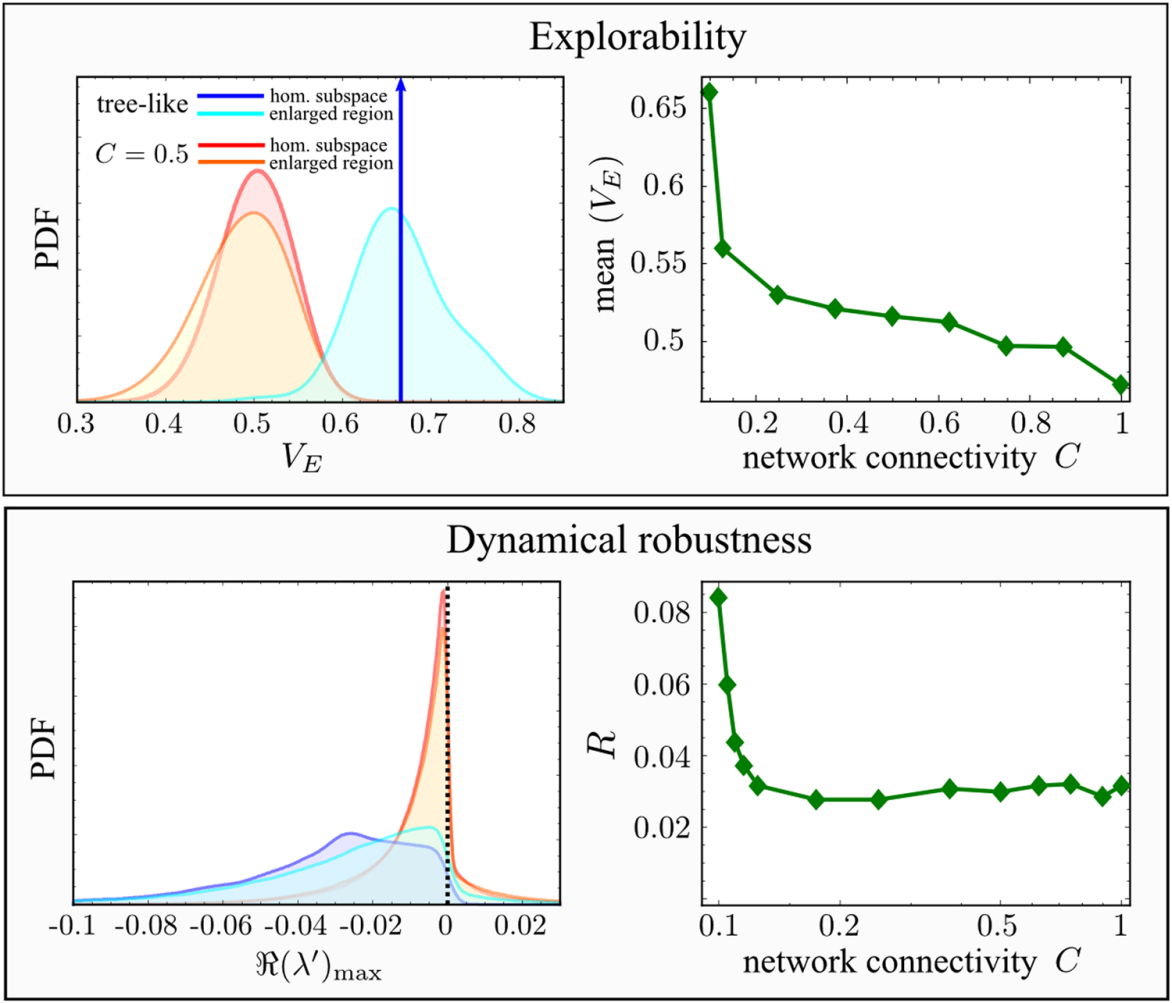
Explorability and dynamical robustness for different connectivities. (Top left) Probability distribution functions (PDF) of the explorability *V*_*E*_ for the optimal tree-like graphs and networks with *C* = 0.5 obtained by adding extra links with random (uniformly distributed) locations and weights ${\varepsilon }_{ij}$ taken from a zero-mean Gaussian distribution with the standard deviation ${\sigma }_{\varepsilon }=0.1$, for a network size *S* = 20. First, we computed *V*_*E*_ in the simple setting of uniform concentrations and growth rates, i.e. ${x}_{i}^{\ast }={x}^{\ast }$ independent of *i* and ${\alpha }_{i}\,=\,1$. The explorability ${V}_{E}\,=\,\mathrm{2/3}$ of the tree-like network (calculated analytically - solid blue line) is larger than the one corresponding to graphs with higher density (red curves were computed by taking 10^3^ independent realizations of the added links). Similar results hold also in the more general setting of non-uniform concentrations and growth rates (sampled from Gaussian distributions with zero mean and standard deviation ${\sigma }_{x}={\sigma }_{\alpha }\,=\,0.1$, see Methods). Even with such a variability, the tree-like network (cyan curve) generally exhibits higher values of the explorability than more dense networks (orange curve for *C* = 0.5). (Top right) Mean value of the explorability (computed from the PDF of *V*_*E*_) as a function of the connectivity in the homogeneous case (${x}_{i}^{\ast }={x}^{\ast }$ and ${\alpha }_{i}=1$). (Bottom left) Given ${w}_{ij}^{{\rm{edge}}}$ (see Fig. 2C), we calculated the Jacobian matrix of the perturbed dynamics ${{J}^{{\rm{^{\prime} }}}}_{ij}={\xi }_{i}^{{\rm{^{\prime} }}}{w}_{ij}^{{\rm{e}}{\rm{d}}{\rm{g}}{\rm{e}}}$. The corresponding eigenvalue with the largest real part ($\Re {(\lambda ^{\prime} )}_{\max }$) gave the degree of stability for the new fixed point, from which we measured the dynamical robustness of the system (see Methods). We generated 10^3^ configurations of ${\varepsilon }_{ij}$’s as done for the top panel (${\varepsilon }_{ij}=0$ corresponds to a tree-like network), and for each one, 10^3^ values of $\xi ^{\prime}$ (encoding the perturbation of the dynamics) from a uniform distribution in $\mathrm{[0,1]}$, and then computed the system response through the distribution $P(\Re {(\lambda ^{\prime} )}_{\max }|C)$. Both homogeneous and non-homogeneous settings were analyzed for the tree-like network and $C=0.5$ (same color code as for the top left panel). In all cases, we found that increasing the network connectivity shifted the distribution of ${\rm{\Re }}({\lambda }_{max}^{{\rm{^{\prime} }}})$ towards the less stable region. Qualitatively similar results were obtained for larger values of ${\sigma }_{\varepsilon }$, ${\sigma }_{x}$ and ${\sigma }_{\alpha }$ (see Supplementary Information). (Bottom right) Dynamical robustness *R* (estimated as the value of $\Re {(\lambda ^{\prime} )}_{\max }$ located at the fifth percentile of the distribution, $R=-\Re {(\lambda ^{\prime} )}_{\max }^{5th}$) as a function of the connectivity in the homogeneous case (${x}_{i}^{\ast }={x}^{\ast }$ and ${\alpha }_{i}=1$), plotted in linear-log scale. Parameters *S* and ${\sigma }_{\varepsilon }$ are set as in the top panels.

Numerical results are represented in Fig. 3 (top left panel), illustrating that the explorability of the optimal tree-like network is indeed statistically higher than the one for denser networks. Furthermore, the average explorability decreases as the connectivity of the interaction network increases (Fig. 3, top right panel). We were able to prove this result for some particular topologies of small networks, for which the explorability can be calculated analytically (see Supplementary Information). In conclusion, our results suggest that, on average, explorability decays with the connectivity of the system, and therefore, sparse networks generally lead to higher values of explorability.

### Dynamical robustness

Another crucial property of complex interacting systems is their robustness to perturbations^31,32^, Understanding the role of network architecture in the stability of a system with many degrees of freedom is an important challenge, since it impacts on our capacity both to prevent system failures and to design more robust networks to tolerate perturbations to the system dynamics.

The standard measure of stability (known as asymptotic resilience in ecology^33,34^,) is defined as the capacity of the system to return to the original stationary state after a perturbation of it, ${{\bf{x}}}^{\ast }+\delta {\bf{x}}$, while the dynamics is kept fixed. Let us note that our definition of explorability already takes into account this kind of stability, i.e. explorability is defined only for stable system dynamics.

Alternatively, we can study how the stability of the system is modified as a result of a perturbed dynamics, $\dot{{\bf{x}}}=(G+\delta G)(F+\delta F)({\bf{x}})$, where $\delta G$ and $\delta F$ represent the perturbations with respect to the original dynamics. This can be understood as including further non-linear effects that were not present before. As a consequence of this kind of perturbation, *both the original stationary states and their degree of stability are modified*. We then quantify the capacity of the system to re-organize after a perturbation of the dynamics such that the new stationary state of the system is close to the original one and still stable. We refer to this as the *dynamical robustness* of the system (to avoid confusing this new measure of stability with the standard resilience).

We found the pleasing result that the Jacobian matrix evaluated at the new stationary state, ${J}_{ij}^{{\rm{^{\prime} }}}$, retained a similar form than for the original dynamics, i.e. ${J}_{ij}^{^{\prime} }={\xi }_{i}^{^{\prime} }{w}_{ij}$, where $\xi ^{\prime}$ depends on the specific details of the perturbed dynamics (see Supplementary Information). Focusing on the worst case, which corresponds to marginally stable fixed points for which $\Re {(\lambda )}_{\max }\,=\,0$ (denoted by ${w}^{{\rm{edge}}}$), we perturbed the dynamics and computed the maximum real part of the eigenvalues of ${J}_{ij}^{{\rm{^{\prime} }}}$ at the new stationary point, $\Re {(\lambda ^{\prime} )}_{\max }$. For a given deterministic perturbation of the dynamics ${\xi }^{{\rm{^{\prime} }}}$ and topology, $\Re {(\lambda ^{\prime} )}_{\max }$ can be taken as a measure of the dynamical robustness (see Fig. 2, panel C). Since we wanted to keep the analysis as general as possible, we studied the dynamical robustness against random perturbations of the dynamics (generated from a distribution $P(\xi ^{\prime} )$). In particular, for each connectivity *C*, we fixed the additional links to ${\varepsilon }_{ij}$ and then looked for the set of matrices ${w}^{{\rm{edge}}}(\{{\varepsilon }_{ij}\})$ at the edge of stability (for which, by definition, $\Re {(\lambda )}_{\max }\,=\,0$). Taking different realisations of ${\varepsilon }_{ij}$ and ${\xi ^{\prime} }_{i}$, we compared the distributions $P(\Re {(\lambda ^{\prime} )}_{\max }|C)$ (see Fig. 2). We can then define a statistical measure *R* of the dynamical robustness by taking the value of $-\Re {(\lambda ^{\prime} )}_{\max }$ located at the fifth percentile, $R=-\Re {(\lambda ^{\prime} )}_{\max }^{5th}$. In this way, we have an indicator of how much stability could be gained under random perturbations of the dynamics. Other choices of this measure can be taken, e.g. based on the 10th, 20th and 50th percentiles, leading qualitatively to the same conclusion. However, lower percentile values generally enhance the differences between topologies (see Supplementary Information).

The bottom left panel of Fig. 3 shows the histogram of $\Re {(\lambda ^{\prime} )}_{{\rm{\max }}}$ for different connectivities. Again, it can be seen that the case of a tree-like network leads to the best performance, whereby the fixed point of the perturbed dynamics generally becomes stable, i.e. $\Re {(\lambda ^{\prime} )}_{{\rm{\max }}}$ is negative, and the modulus reaches larger values than in the corresponding case of networks with higher connectivity. Therefore, our analysis shows that sparse tree-like networks have both a larger explorability and a larger dynamical robustness than random networks with higher connectivity.

### Optimisation approach

We then went one step further and compared the explorability and dynamical robustness of tree-like networks with graphs constructed via an optimisation process rather than randomly generated.

In the optimisation, weight values ${\varepsilon }_{ij}$ were changed accordingly to a stochastic hill climbing algorithm^35^, whereas their locations were kept fixed. Introducing a random Gaussian perturbation with zero mean and standard deviation $\delta \varepsilon$ in all ${\varepsilon }_{ij}$, the new configuration was accepted if the quantity to optimise (either ${V}_{E}$ or *R*) increased, and the process was iterated for *T* time steps. Our results are robust for different choices of $\delta \varepsilon$ in the range [10^−3^, 10^−1^]. We restricted our analysis to the homogeneous case (${x}_{i}^{\ast }={x}^{\ast },{\alpha }_{i}=\alpha$), using network sizes of *S* = 10 to facilitate the convergence of the optimisation algorithms. Still, the landscape of ${V}_{E}({\varepsilon }_{ij})$ and $R({\varepsilon }_{ij})$ appeared to be highly irregular with many local minima when increasing the connectivity.

**Figure 4:**
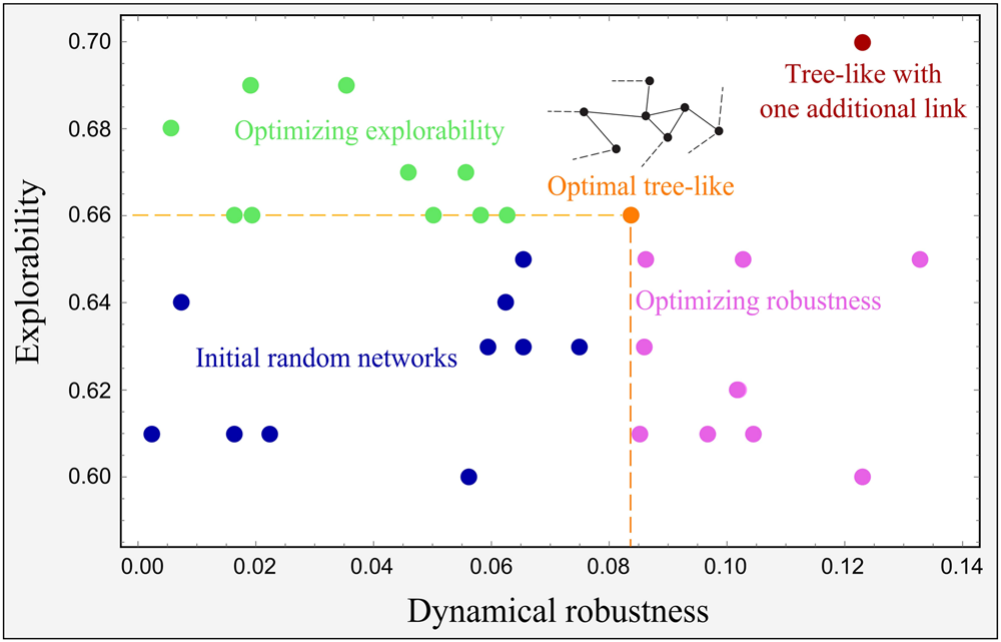
Optimisation of random generated networks of size *S* = 10 and with different connectivities in the range [0.25, 0.75] (blue dots) in the explorability (green dots) and dynamical robustness (magenta dots). The optimal tree-like network (containing *S* edges) is represented by the orange dot. The red dot in the right top corner represents a tree-like network with one additional link (i.e. a network with *S* + 1 edges), obtained using a multi-objective optimization for both the explorability and the dynamical robustness; such a method appeared to be inefficient for higher values of the connectivity. Each network was optimized using a stochastic hill climbing algorithm^35^ with parameters *T* = 200 and $\delta \varepsilon =0.01$.

Figure 4 represents the initial random-generated networks with connectivities in the range $C\in \mathrm{[0.25,}\,\mathrm{0.75]}$ (blue dots) and the corresponding optimised values for the explorability and the dynamical robustness (green and magenta dots, respectively). Qualitatively similar results are found using different schedules of the simulated annealing algorithm^36^. The explorability reached for networks with connectivities larger than the one for tree-like networks turned out to be very close to the corresponding value for the optimal tree-like topology. However, in general, such networks exhibited low values of dynamical robustness. Similarly, when optimising the dynamical robustness we ended up with values very close to that of the optimal tree-like network, but remarkably, without improving the explorability. Indeed, in all the cases that we have analyzed, during the optimization of one property, the other one either remained unaltered or decreased. However, for denser networks the optimization becomes harder due to the presence of many local minima and it was rather difficult to draw general conclusions on how the non-optimized property behaves with the connectivity.

We also implemented a multi-objective optimisation algorithm, in which a perturbation in ${\varepsilon }_{ij}$ was only accepted if it increased simultaneously both the explorability and the dynamical robustness of the network. However, this method worked only for tree-like networks with one or two additional links (see red dot in Fig. 4), while it was totally inefficient for more dense structures. Slightly better (but still suboptimal) results were obtained when both tasks were optimised one at a time in alternating periods.

In conclusion, sparse networks provide quasi-optimal values for both the explorability and dynamical robustness without fine-tuning many of the interaction strengths.

### Self-similarity

Finally, we proved that the property of sparsity is self-similar, since on aggregating sparse interacting communities, we obtained larger sparse communities (see Supplementary Information for details). For example, joining two networks with a tree-like topology using a single link led again to a network with a tree-like topology. Similarly, if sparse networks with *S* nodes have $aS-b$ links, with *a* and *b* integer constants, then joining two such networks with *S* and S′ nodes using *b* links leads again to a sparse network with $a(S+S^{\prime} )-b$ links. Therefore, the optimal features of sparsity are conserved on assembling or disassembling processes, thereby avoiding any drastic change in the stability^37^.

## Discussion

Our approach provides a theoretical insight into why sparsity is an observed common feature in living interacting systems. Sparse networks generally offer optimal values of both explorability and dynamical robustness, whereas denser networks can only perform better if interactions are selectively tuned. Nevertheless, we observed that finding dense optimal networks with higher values of both explorability and dynamical robustness was barely feasible due to the multiplicity of the parameters that must be simultaneously tuned. Moreover, typically, the final networks have values of explorability and dynamical robustness comparable to those achievable for tree-like networks structures without the need to tune any parameters.

The results presented support the idea that sparsity is an emergent pattern of living interaction networks and this has implications for the understanding of the relationship between stability and complexity in real ecosystems. Indeed, sparsity may play a key role in the resolution of the so-called complexity-stability paradox^38,39^, in which highly biodiverse ecosystems will probably be unstable. The essence of the argument^38,39^, can be summarised as follows. The linearised dynamics for the population density around a stationary state depends on what is known as the community matrix, *M*. If all the eigenvalues of *M* have negative real parts, then the stationary point is also stable against small perturbations of the stationary populations. A null model corresponds to assume that *M* is a random matrix with diagonal elements (the self-interactions) equal to −*d* < 0, whereas the off-diagonal elements are zero with probability 1 − *C* and with probability *C* are drawn from a probability distribution with zero mean and variance ${\sigma }^{2}$. Under this null hypothesis, one finds (see^38,39^, and references therein for rigorous results) that the stationary point is unstable with probability 1 if $\sigma \sqrt{CS} > d$, where *S* is the number of species in the ecosystem (a measure of its biodiversity). This result holds if *S* is assumed to be large enough. Thus if $\sigma$ and *d* do not have a peculiar scaling with the network size, highly complex ecosystems (i.e. with high *CS*) are not stable: a prediction in contradiction to empirical data^11,37,40^, However if the interaction network is sparse, i.e. $C\sim 1/S$, the above inequality becomes independent of *S* and the stability of the ecosystem is not threatened by high biodiversities: sparsity in ecological interaction networks allows for stable large living interacting systems^11,19,37^, As a matter of fact, such a scaling relationship is supported by the empirical observation (see Fig. 1).

Recent theoretical findings show that an increase in the interconnectivity between multiple systems composed themselves of interacting units can have a strong impact on the vulnerability of the whole system^41^. In the same vein, our results provide a theoretical understanding of this feature. We suggest that sparsity is a key feature allowing living systems to be poised in a state that confers both robustness and adaptability (explorability) to best cope with an ever-changing environment and to promptly react to a wide range of external stimuli and to resist to perturbations.

Our ideas could be applied to understand the emergence of sparsity in some non-biological systems, as it has been empirically observed in human-built networks^42^. The underlying hypothesis of our approach is that living system interactions, unlike physical interactions (e.g. electromagnetic interactions among charged particles), can evolve/adapt to turn themselves on or off. The same idea holds for many non-biological networks, i.e. interactions can be selective and change in time, although the equations governing the underlying dynamics can be unknown.

Finally, we stress that our results do not depend on the specific details of the system and thus can be applied in many other fields. For example, a possible application might be in the design of artificial learning machines, such as deep neural networks^43,44^, There is mounting evidence that deep learning often finds solutions with good generalisation properties^43,45^, and it has been shown recently^46^ that to achieve such a good performance, it is crucial to have regions of the optimisation landscape that are both robust and accessible, independent of the particular task or of the training data set. On the other hand, maximisation of computation efficiency is a crucial point when designing learning machines: deep networks are very dense as each node is connected to all other nodes of the adjacent layers^44^, which makes multilayer neural networks computationally hard to train. Our solution suggests that designing sparse neural networks will increase the explorability of the system while improving the convergence and robustness properties of the existing optimisation algorithms.

## Methods

### Measuring explorability

The explorability of a tree-like network with *S* links (*C* = 2/5) can be found by studying the following inverse problem: by fixing the parameters ${\alpha }_{i}$ and moving along the space of fixed points, one can retrieve the non-zero *S* values of *w*_*ij*_ according to the fixed point equation ${\sum }_{j}{w}_{ij}{x}_{j}^{\ast }=-{\alpha }_{i}$ and this can then be used to check the stability of the associated fixed point ${{\bf{x}}}^{\ast }$. The solution exists if for each node, *i*, there is at least a node *j*, such that ${w}_{ij}\ne 0$ (see Supplementary Information). The same procedure can be applied to the more general case where extra links are added to the network, each one with a given fixed strength ${\varepsilon }_{ij}$ (see Supplementary Information for more detail).

### Increasing heterogeneity

We also calculated the explorability increasing the heterogeneity in both the dynamics parameter (${\alpha }_{i}$) and the fixed points (${x}_{i}^{\ast }$) that were sampled: we took random realizations of ${\alpha }_{i}=\alpha +{q}_{i}$, where the *q*_*i*_’s were independent Gaussian random variables with zero mean and standard deviation ${\sigma }_{\alpha }$ (as for the simple case, we set *α* = 1); for each realization, we also enlarged the region explored around the homogeneous state by taking different realizations of ${x}_{i}^{\ast }={x}^{\ast }+{p}_{i}$ (where the *p*_*i*_’s are distributed as the ${q}_{i}$’s with standard deviation ${\sigma }_{x}$). Varying the value of ${x}^{\ast }$, we counted *all* the fixed points at the edge of stability (within a small error $|\Re {(\lambda )}_{{\rm{\max }}}| < {10}^{-2}$), and for each one we evaluated ${V}_{E}=1-{\sum }_{i}{x}_{i}^{\ast }/S$ as the most straightforward generalization of our previous definition of *V*_*E*_; then, we constructed the histogram of *V*_*E*_. The curves in Fig. 3 for the case with heterogeneity were obtained using 10^2^ independent realizations of ${\varepsilon }_{ij}$, and, for each one, 10 realizations of *p*_*i*_ and *q*_*i*_, respectively.

## Electronic supplementary material

Supplementary Information

## References

[1] Liu Y-Y, Slotine J-J, Barabási A-L (2011). Controllability of complex networks. Nature.

[2] Nacher, J. C. & Akutsu, T. Structural controllability of unidirectional bipartite networks. *Scientific reports* **3** (2013). 10.1038/srep01647 PMC3622082 23571689

[3] Babu MM, Luscombe NM, Aravind L, Gerstein M, Teichmann SA (2004). Structure and evolution of transcriptional regulatory networks. Current opinion in structural biology.

[4] Milo R (2002). Network motifs: simple building blocks of complex networks. Science.

[5] Banavar JR, Damuth J, Maritan A, Rinaldo A (2002). Ontogenetic growth (communication arising): modelling universality and scaling. Nature.

[6] Banavar JR, Maritan A, Rinaldo A (1999). Size and form in efficient transportation networks. Nature.

[7] West GB, Brown JH (2004). Life’s universal scaling laws. Physics today.

[8] Stanley H (2000). Scale invariance and universality: organizing principles in complex systems. Physica A: Statistical Mechanics and its Applications.

[9] Stanley H (1996). Scaling and universality in animate and inanimate systems. Physica A: Statistical Mechanics and its Applications.

[10] Bascompte J, Jordano P, Melián CJ, Olesen JM (2003). The nested assembly of plant–animal mutualistic networks. Proceedings of the National Academy of Sciences.

[11] Suweis S, Simini F, Banavar JR, Maritan A (2013). Emergence of structural and dynamical properties of ecological mutualistic networks. Nature.

[12] Dunne JA, Williams RJ, Martinez ND (2002). Food-web structure and network theory: the role of connectance and size. Proceedings of the National Academy of Sciences.

[13] Fraser LH (2015). Worldwide evidence of a unimodal relationship between productivity and plant species richness. Science.

[14] Pascual, M. & Dunne, J. A. *Ecological networks: linking structure to dynamics in food webs* (Oxford University Press, 2006).

[15] Kunin WE, Gaston KJ (1993). The biology of rarity: patterns, causes and consequences. Trends in Ecology & Evolution.

[16] Barzel B, Barabási A-L (2013). Universality in network dynamics. Nature physics.

[17] Garlaschelli D, Caldarelli G, Pietronero L (2003). Universal scaling relations in food webs. Nature.

[18] Suweis, S., Grilli, J., Banavar, J. R., Allesina, S. & Maritan, A. Effect of localization on the stability of mutualistic ecological networks. *Nature communications* **6** (2015). 10.1038/ncomms10179 PMC4703855 26674106

[19] Grilli, J. *et al*. Feasibility and coexistence of large ecological communities. *Nature Communications* **8** (2017). 10.1038/ncomms14389 PMC5333123 28233768

[20] Bollobás, B. *Modern graph theory*, vol. 184 (Springer Science & Business Media, 2013).

[21] Bialek, W. *Biophysics: searching for principles* (Princeton University Press, 2012).

[22] Stone, L. The google matrix controls the stability of structured ecological and biological networks. *Nature Communications* **7** (2016). 10.1038/ncomms12857 PMC5056432 27687986

[23] Ackland G, Gallagher I (2004). Stabilization of large generalized Lotka-Volterra foodwebs by evolutionary feedback. Physical review letters.

[24] Coyte KZ, Schluter J, Foster KR (2015). The ecology of the microbiome: Networks, competition, and stability. Science.

[25] Bashan A (2016). Universality of human microbial dynamics. Nature.

[26] Hirafuji, M., Tanaka, K. & Hagan, S. Lotka-volterra machine for a general model of complex biological systems. In *Computer Aided Control System Design, 1999. Proceedings of the 1999 IEEE International Symposium on*, 516–521 (IEEE, 1999).

[27] Draghi JA, Parsons TL, Wagner GP, Plotkin JB (2010). Mutational robustness can facilitate adaptation. Nature.

[28] Wagner A (2012). The role of robustness in phenotypic adaptation and innovation. Proceedings of the Royal Society of London B: Biological Sciences.

[29] Schreier H, Soen Y, Brenner N (2016). Exploratory adaptation in large random networks. arXiv preprint arXiv.

[30] Pinho R, Borenstein E, Feldman MW (2012). Most networks in wagner’s model are cycling. PloS one.

[31] Nelson DR, Adger WN, Brown K (2007). Adaptation to environmental change: contributions of a resilience framework. Annual review of Environment and Resources.

[32] Suweis S, Carr JA, Maritan A, Rinaldo A, D’Odorico P (2015). Resilience and reactivity of global food security. Proceedings of the National Academy of Sciences.

[33] Arnoldi J-F, Loreau M, Haegeman B (2016). Resilience, reactivity and variability: A mathematical comparison of ecological stability measures. Journal of theoretical biology.

[34] Gao J, Barzel B, Barabási A-L (2016). Universal resilience patterns in complex networks. Nature.

[35] Russell, S. J., Norvig, P., Canny, J. F., Malik, J. M. & Edwards, D. D. *Artificial intelligence: a modern approach*, vol. 2 (Prentice hall Upper Saddle River, 2003).

[36] Kirkpatrick S, Gelatt CD, Vecchi MP (1983). Optimization by simmulated annealing. science.

[37] McCann KS (2000). The diversity–stability debate. Nature.

[38] May RM (1972). Will a large complex system be stable?. Nature.

[39] Allesina S, Tang S (2012). Stability criteria for complex ecosystems. Nature.

[40] Azaele S (2016). Statistical mechanics of ecological systems: Neutral theory and beyond. Reviews of Modern Physics.

[41] Vespignani A (2010). Complex networks: The fragility of interdependency. Nature.

[42] Blagus N, Šubelj L, Bajec M (2012). Self-similar scaling of density in complex real-world networks. Physica A: Statistical Mechanics and its Applications.

[43] LeCun Y, Bengio Y, Hinton G (2015). Deep learning. Nature.

[44] Goodfellow, I., Bengio, Y. & Courville, A. *Deep Learning* (MIT Press, 2016).

[45] Kashtan N, Alon U (2005). Spontaneous evolution of modularity and network motifs. Proceedings of the National Academy of Sciences of the United States of America.

[46] Baldassi C (2016). Unreasonable effectiveness of learning neural networks: From accessible states and robust ensembles to basic algorithmic schemes. Proceedings of the National Academy of Sciences.

