# Supplementary material for: Explorability and the origin of network sparsity in living systems
Source: Sci Rep. 2017 Sep 26;7:12323. doi: 10.1038/s41598-017-12521-1 (PMC5615038; doi:10.1038/s41598-017-12521-1)
Supplement: Supplementary file 1 — Supplementary Information [file 41598_2017_12521_MOESM1_ESM.pdf]

Explorability and The Origin of Network Sparsity in Living Systems

(Supporting Information)

D. M. Busiello, S. Suweis, J. Hidalgo and A. Maritan*
Dipartimento di Fisica 'G. Galilei' & CNISM, INFN,
Università di Padova, Via Marzolo 8, 35131 Padova, Italy
*amos.maritan@pd.infn.it

August 2, 2017

Contents

1 Experimental data **2**

2 Measuring explorability **4**
2.1 Analytical solution for a tree-like network in the uniparametric case 4
2.2 Numerical results increasing the number of links 6
2.2.1 Defining a threshold of stability 8
2.3 Introducing variability in the explored region 9
2.4 From a Lotka-Volterra model to a more general dynamics 12

3 Dynamical robustness **13**
3.1 Perturbing the dynamics 13
3.2 Including additional information in the perturbed Jacobian matrix 13
3.3 Measuring dynamical robustness 16

4 From sparse interaction matrices to community structures **17**

1 Experimental data

The following table lists all experimental datasets used in Figure 1 of the Main Text, indicating details of the corresponding networks.

Type of network	Name	Nodes	Edges	Connectivity
Neural network	Brain C.Elegans [14]	252	509	0.00805
Gene-regulation	TRN Yeast-1 [14]	4441	12873	0.000653
	TRN Yeast-2 [14]	688	1079	0.00228
	TRN E.Coli-1 [14]	1550	3340	0.00139
	TRN E.Coli-2 [14]	418	519	0.00298
	TRN S.Cerevisiae [16]	723	2158	0.00413
Trust	College student [14]	32	96	0.0968
	Prison inmate [14]	67	182	0.0411
Protein interaction	Drugs-Targets [16]	1282	3186	0.00194
	ncRNA human [16]	188	498	0.0142
	ncRNA 6-organisms [16]	523	1294	0.00474
Metabolic	E.Coli [14]	2275	5763	0.00111
	S.Cerevisiae [14]	1511	3833	0.00168
	C.Elegans [14]	1173	2864	0.00208
FW Host-Parasite	Altuda 1979 [11]	47	122	0.0564
	Marathon 1979 [11]	78	346	0.0576
	Britain 1991 [12]	94	232	0.0265
	Finland 1991 [12]	69	190	0.0405
FW Plants-Herbivors	Cold lake [13]	50	182	0.0743
	Lake of the woods [9]	175	768	0.0252
	Lake Huron [3]	130	632	0.0377
	Smallwood reservoir [7]	31	106	0.114
	Parsnip river [2]	66	228	0.0531
	McGregor river [2]	70	316	0.0654
Mutualistic	Cordn del Cepo [19]	185	722	0.0212
	Cordn del Cepo [19]	107	392	0.0346
	Cordn del Cepo [19]	61	162	0.0443
	Central New Brunswick [19]	114	334	0.0259
	Princeton [19]	26	62	0.0954
	Mount Missim [19]	38	204	0.145
	Caguana [19]	39	128	0.0864
	Cialitos [19]	52	174	0.0656
	Cordillera [19]	36	94	0.0746
	Fronton [19]	34	96	0.0856
	Pikes Peak [19]	371	1846	0.0134
	Tropical rainforest [19]	77	268	0.0458
	Hickling [19]	78	292	0.0486
	Shelfanger [19]	52	170	0.0641
	Tenerife [19]	49	212	0.0901
	Latnjajaure [19]	142	484	0.0242
	Zackenberg [19]	107	912	0.0804
	Mauritius Island [19]	27	104	0.148
	Mtunzini [19]	24	182	0.330
	Santa Genebra Reserve [19]	23	62	0.122

	Santa Genebra Reserve [19]	62	238	0.0629
	North Negros Forest Reserve [19]	63	394	0.101
	Doñana National Park [19]	205	824	0.0197
	Hazen Camp [19]	110	358	0.0299
	Hato Ratn [19]	31	182	0.196
	Snowy Mountains [19]	127	538	0.0336
	Campeche State [19]	30	84	0.0965
	Hazen Camp [19]	111	380	0.0311
	Ashu [19]	770	2386	0.00403
	Kuala Lompat [19]	84	890	0.128
	Gabon [19]	25	122	0.203
	CMBRS [19]	62	124	0.0328
	Laguna Diamante [19]	66	166	0.0387
	Rio Bianco [19]	95	250	0.0280
	Bristol [19]	104	598	0.0558
	Melville Island [19]	29	76	0.0936
	Monteverde [19]	208	1322	0.0307
	North Carolina [19]	57	286	0.0896
	Galapagos [19]	159	408	0.0162
	Nava Correhuelas [19]	56	214	0.0695
	Nava Noguera [19]	44	174	0.0920
	Flores [19]	22	60	0.130
	Hestehaven [19]	49	124	0.0527
	Garajonay [19]	84	290	0.0415
	KwaZulu-Natal region [19]	64	198	0.0491
	Jamaica [19]	97	356	0.0382
	Arhur's pass [19]	78	240	0.0400
	Cass [19]	180	748	0.0232
	Craigieburn [19]	167	692	0.0250
	Daphn [19]	797	5866	0.00925
	Guarico State [19]	86	218	0.0298
	Canaima National Park [19]	97	312	0.0335
	Yakushima Island [19]	33	52	0.0492
	Brownfield [19]	40	130	0.0833
	Ottawa [19]	47	282	0.130
	Chilo [19]	156	624	0.0258
	Tropical rainforest [19]	62	414	0.109
	Intervales and Saibadela [19]	315	2106	0.0213
	Great Britain [19]	23	60	0.118

2 Measuring explorability

We detail the method developed in the main text to estimate the explorability of a given topology. The section is organized as follows: *i)* We solve analytically the uniparametric case $\alpha_i = \alpha$, computing the volume of feasible and stale fixed points as a function of the threshold of stability, moving along the bisector $x_i^* = x^*$. By investigating the tree-like structures, we find an optimal tolopology that maximizes the explorability. *ii)* We increase the connectivity by including additional links to the optimal tree-like network, whose weights are fixed. In the case in which we add a few edges, we are able to find some particular interaction matrices which enlarge the value of explorability respect to the sparsest tree-like case. *iii)* We increase the explored region around the bisector and study the stability of the attractors as a function of the variability introduced. *iv)* We analyze the explorability for a more generic case of non-linear dynamics.

2.1 Analytical solution for a tree-like network in the uniparametric case

We start considering the simplest case of a $S \times S$ matrix w with S off-diagonal links, fixing the diagonal elements to $w_{ii} = -1$ [15, 17]. For a given value of x_i^* and α_i for each i, edges weights can be determined by solving the fixed point equation:

$$\sum_j w_{ij} x_j^* = -\alpha_i. \tag{S1}$$

Eq. (S1) has a unique solution if w has one non-diagonal entry per row, which means that the network must have one single loop, beyond the S self loops due to the diagonal entries. In this situation the interaction matrix is sparse, and its connectivity C is equal to:

$$C = \frac{\textit{number of links}}{\textit{matrix dimension}} = \frac{2}{S} \tag{S2}$$

In order to evaluate the explorability of this network, we first investigate the bisector of the attractor space, setting all the components of a fixed point equal to x^*. We also fix model parameters to $\alpha_i = \alpha$ for sake of simplicity. In this case, the weights w of the S off-diagonal links in the interaction matrix are:

$$w = \frac{x^* - \alpha}{x^*} \tag{S3}$$

Eq. (S3) does not depend on the specific position of the S links. In order to analyze the stability of a fixed point $\mathbf{x}^*$, we have to write the characteristic polynomial of the Jacobian matrix J and seek for the real part of its principal eigenvalue, $\Re(\lambda)_{\max}$. Note that J has the same topology of w, with $-x^*$ as diagonal elements and wx^* as off-diagonal

elements. One can express the determinant of $J^{(\lambda)} = J - \lambda \mathbf{1}$ using the Grassmann variables $\{\chi_i, \chi_i^*\}_{i=1}^S$ [4] in terms of the following Gaussian integral:

$$\det J^{(\lambda)} = \int d\chi_1 ... d\chi_S d\chi_1^* ... d\chi_S^* \exp\left(\chi_k J_{kl}^{(\lambda)} \chi_l^*\right) \tag{S4}$$

Using the properties of the Grassmann variables [4], from Eq. (S4) we can state the following operative rules to derive the characteristic polynomial of $J^{(\lambda)}$:

1. Represent the interaction matrix as a graph with a fixed number of loops.
2. Associate to each self-loop a weight $-x^* - \lambda$ and to the links a weight $x^* - \alpha$.
3. Consider all possible combinations of links forming loops reaching once every node in the graph.
4. The weight of each of these combinations is obtained by multiplying the weights associated to the links and a factor-1 for each loop in the combination under consideration.
5. Sum up each of these contributions to obtain the characteristic polynomial.

Using this method, it is easy to demonstrate that, for the simple uniparametric case of a tree-like network with one single loop, the characteristic polynomial is:

$$\det J^{(\lambda)} = (x^* + \lambda)^{S-l}[(x^* + \lambda)^l - (x^* - \alpha)^l], \tag{S5}$$

where $l > 1$ is the number of links of the loop. The real part of the corresponding roots are:

$$\Re(\lambda_k) = -x^* + (x^* - \alpha)\cos\left(\frac{2k\pi}{l}\right) \qquad k = 1, ...l. \tag{S6}$$

By imposing that the maximum of these values to be negative, which guarantees that $\mathbf{x}^* = (x^*, \ldots, x^*)$ is a stable fixed point, we obtain the following inequalities:

$$\begin{aligned} x^* > x_c^* = \frac{\alpha}{2} \qquad & \text{if} \quad l = 2n \\ x^* > x_c^* = \frac{\alpha}{3} \qquad & \text{if} \quad l = 3 \\ x^* > x_c^* = \frac{\alpha}{1 + \phi(n)} \qquad & \text{if} \quad l = 2n + 3 \end{aligned} \tag{S7}$$

where n is a positive integer and $\phi(n) = -\left(\cos\left(\frac{2(n+1)\pi}{2n+3}\right)\right)^{-1}$. From equation (S7) we find that a network with one loop of length 3 provides the lowest x_c^*, and therefore maximizes the volume of feasible and stable attractors.

The threshold of stability x_c^* can be used as a proxy for the volume of explorability; the larger the value of x_c^*, the smaller the explorability V_E. As we are only interested in comparing the explorability of different networks, one possibility is to take $V_E = V_0 - x_c^*$, with V_0 a constant value that has to be large enough to avoid negative values of V_E. With this definition, $V_E = V_0 - \frac{1}{3}\alpha$ for the tree-like network.

On the other hand, α is a parameter of the dynamics (e.g. the species growth rates), that can be set to $\alpha = 1$ without loss of generality (this only sets a time-scale, although rescaling α without rescaling the weights w_{ij} changes the location of the attractors).

Unless otherwise specified, in what follows we set $V_0 = \alpha = 1$, which leads to $V_E = \frac{2}{3}$ for the optimal tree-like network.

2.2 Numerical results increasing the number of links

Starting from the solution just found, i.e. the optimal tree-like network with a single loop of length 3, we introduce additional weighted links. If in the tree-like network, the link leaving node i and ending at node j is missing, we may add it with its weight denoted by ϵ_{ij}. Following the method described in the previous section, we derive analytically the characteristic polynomial of the Jacobian matrix. Because this expression clearly depends on the positions and weights of the additional links, an exhaustive search of solutions that improve the explorability of the optimal tree-like network cannot be performed. However, in Fig. S1 we show two particular topologies with $V_E > \frac{2}{3}$.

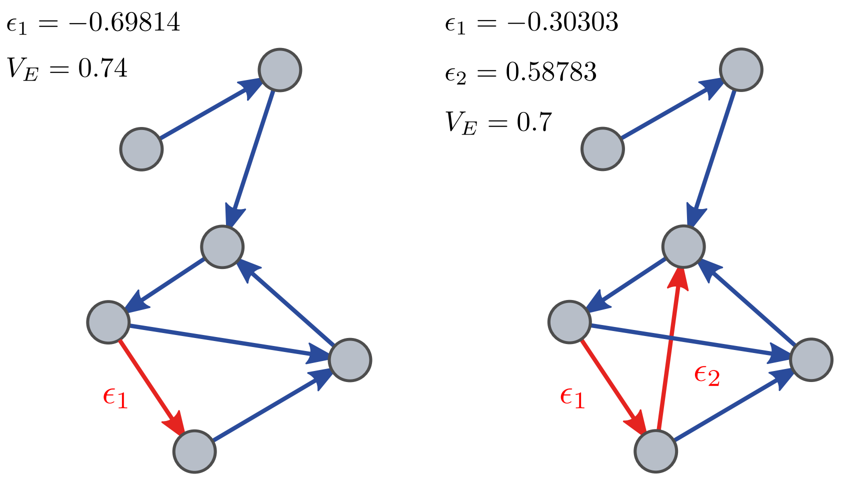

Figure S1: Topologies improving the explorability of the optimal tree-like network: *left)* 2-loop interaction matrix, *right)* 3-loop interaction matrix. We have set $\alpha = 1$ as well as $V_0 = 1$ in the definition of the explorability in terms of the threshold of stability, which leads to $V_E = 2/3$ for the optimal tree-like network.

Although it is possible to improve the explorability of the optimal tree-like network, this becomes harder and harder as the connectivity, C, increases. Indeed, as illustrated in Fig. 3 in the main text, taking random realizations of the added links ϵ_{ij} from a Gaussian distribution with zero mean and standard deviation σ_ϵ and their positions uniformly distributed, we obtain a histogram $P(V_E|C)$ which is shifted to lower values of V_E than the optimal one for a tree-like network. Similar results are shown in Fig. S2 for different values of the σ_ϵ.

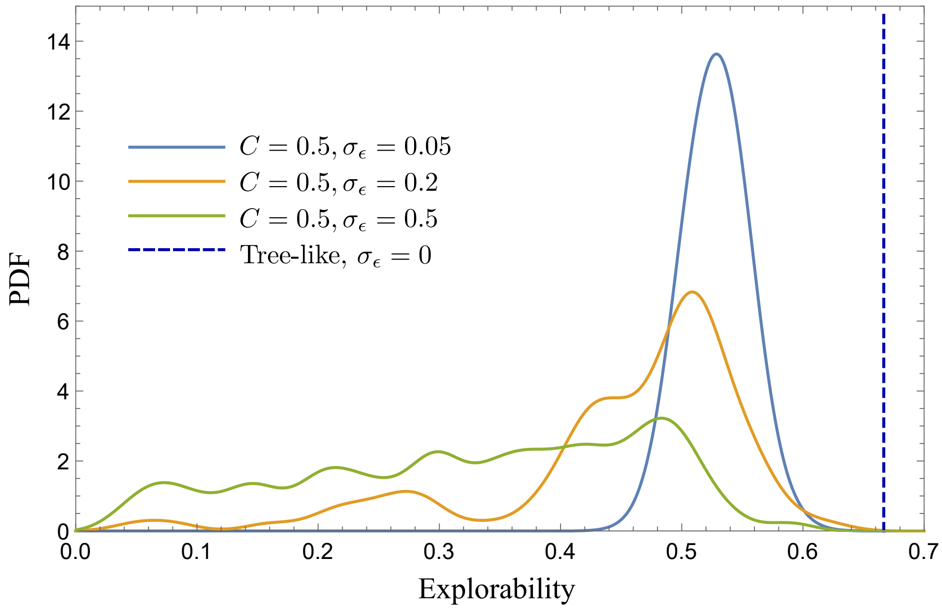

Figure S2: Probability distribution functions (PDF) of the explorability V_E for the optimal tree-like graph and networks with $C = 0.5$ obtained by adding extra links with random (uniformly distributed) locations and weights ϵ_{ij} taken from a zero-mean Gaussian distribution with standard deviation σ_ϵ, for a network size $S = 20$ and $\alpha = 1$, using 10^3 independent realizations of the added links. Increasing the variability of the weights of the added links the explorability generally decreases. We have set $V_0 = 1$ in the definition of the explorability.

2.2.1 Defining a threshold of stability

In the simplest situation in which $x_i^* = x^*$ and $\alpha_i = \alpha$, we observed that, in most cases, $\Re(\lambda)_{\max}$ is positive for small x^* and negative for large x^*, intersecting $\Re(\lambda)_{\max} = 0$ at a single value x_c^*. However, we have observed some singular cases: some of them (less than 1% for $C = 0.5$ and $\sigma_\epsilon = 0.1$) lead to multiple solutions for $\Re(\lambda)_{\max} = 0$ (see Fig. S3). For these cases taking the threshold as the minimum value among all the possible solutions lead to an overestimate of the explorability. As these cases only appear for dense topologies, such an overestimate does not affect our conclusions.

In addition, only for dense networks, we could find some cases (less than 1% for $C = 0.5$ and $\sigma_\epsilon = 0.1$) with no feasible and stable attractors (i.e. with $\Re(\lambda)_{\max}$ always positive), which are not admissible cases in our analysis.

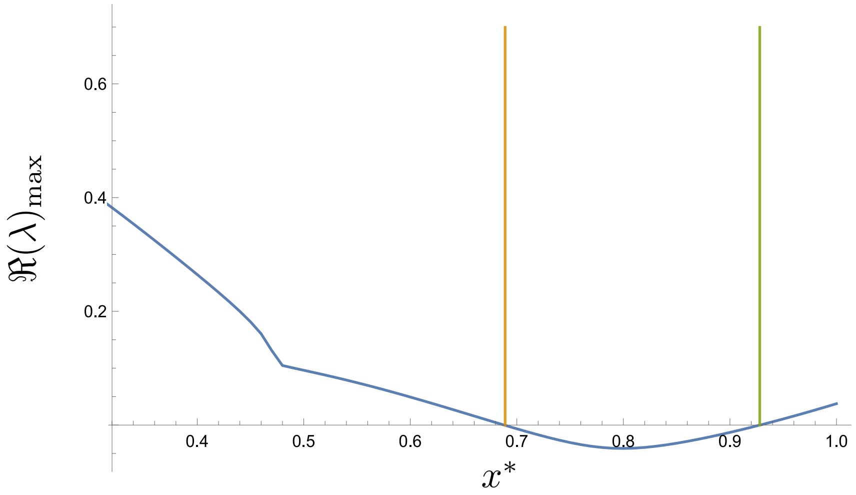

Figure S3: $\Re(\lambda)_{\max}$ as a function of x^* for a particular network with $C = 0.5$ generated using $\sigma_\epsilon = 0.5$ ($\alpha = 1$, $S = 20$). The curve exhibits two solutions for $\Re(\lambda)_{\max} = 0$.

2.3 Introducing variability in the explored region

In the previous sections we carried out an analysis for x_i^* and α_i independent of i, i.e. moving along the bisector of the attractor space in a uniparametric subspace. Here we enlarge the explored region of attractors, by introducing a variability in the fixed point and in the model parameters, as follows:

$$x_i^* = x^* + p_i$$
$$\alpha_i^* = \alpha + q_i$$

for $i = 1, ...S$, where p_i and q_i are independent Gaussian random variables with zero mean and standard deviation σ_x and σ_α, respectively. In this case, we counted all the sampled attractors at the edge of stability (within a small error $|\Re(\lambda)_{\max}| < 10^{-2}$), and for each one we evaluated $V_E = 1 - \sum_i x_i^*/S$ as the most straightforward generalization of the measure of explorability.

Fig. S4 shows, for the case $\sigma_x = \sigma_\alpha$, how the probability of being stable changes for the optimal tree-like network and for the topologies shown in Fig. S1. Notice that such curves become smoother and smoother as the heterogeneity increases. In this case, simulations have been done with $S = 4$, as only links forming loops contribute to the Jacobian (see Section 2.1), and consequently similar results can be expected using larger networks with only 1, 2 and 3 loops.

Finally, similarly to Fig. 3 of the main text, Fig. S5 illustrates the histogram of explorability, $P(V_E|C)$, for different values of $\sigma_x = \sigma_\alpha$, for the tree-like network and for a more dense topology with $C = 0.5$. These results show that the introduction of variability

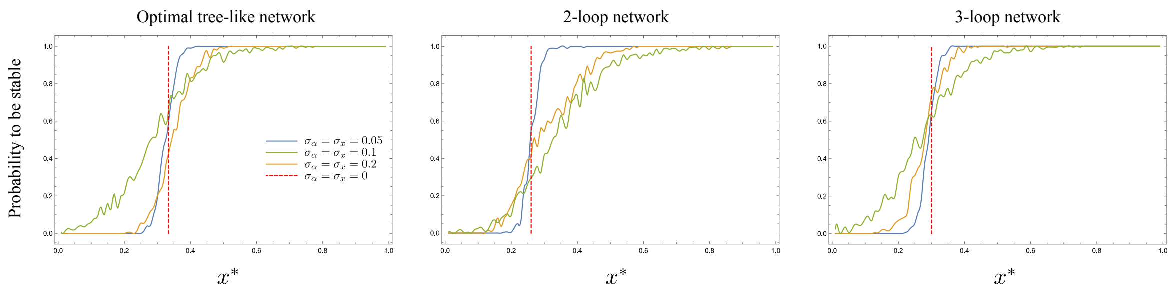

Figure S4: Probability to be stable around the bisector for different network structures and values of heterogeneity. Moving along the bisector $x_i^* = x^*$, we generate 200 independent realization of $(x^* + p_i, \alpha + q_i)$, where p_i and q_i are independent Gaussian variables with zero mean and standard deviation σ_x and σ_α, respectively. We represent the probability of these attractors to be stable as a function of $\bar{x}^* = \sum_i x_i^*/S$ for: *left)* the optimal tree-like network with $S = 4$; *middle)* the 2-loop interaction matrix in Fig. S1 and *right)* the 3-loop network in Fig. S1. Red-dashed line represents the homogeneous case for comparison.

does not change our conclusion, that is sparse networks generally provide larger values of explorability.

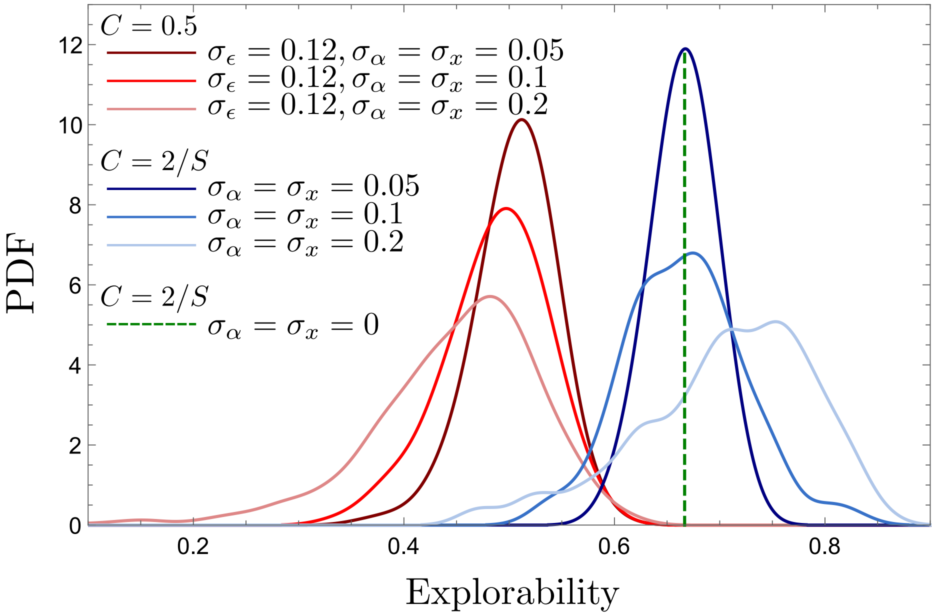

Figure S5: Probability distribution functions (PDF) of the explorability V_E for the optimal tree-like graph and networks with $C = 0.5$. We performed 10^2 independent realizations of the added links ($\epsilon_{ij} = 0$ for the tree-like network), and for each one sampling 10 different choices (p_i, q_i). Network size $S = 20$ and $\alpha = 1$. Variability does not qualitatively influence our main results.

2.4 From a Lotka-Volterra model to a more general dynamics

The results presented in the previous sections implicitly refer to a generalized Lotka-Volterra dynamical model [8, 18, 10, 20, 5, 6, 1]. We can also generalize the dynamics to the form:

$$\dot{x}_i = G_i(x_i) F_i\left(\sum_j w_{ij} x_j\right) \tag{S8}$$

whose fixed point is determined by $F_i(-\alpha_i) = 0$ leading to Eq. (S1) (see Main Text). The Jacobian matrix of such a dynamics is $J_{ij} = G_i(x_i^*) \left.\frac{dF_i}{dz_i}\right|_{-\alpha_i} w_{ij}$, where $z_i = \sum_j w_{ij} x_j$. Since we have no available information of the particular form of $\mathbf{G}$ and $\mathbf{F}$, in the same spirit of May's original contribution [15, 17], we can write the Jacobian matrix as $J_{ij} = \xi_i w_{ij}$, where ξ_i is a random number uniformly distributed between 0 and 1, in analogy with the Lotka-Volterra simple case. Fig. S6 represents the histogram of the explorability for a tree-like matrix and $C = 0.5$, sampling different values of $\boldsymbol{\xi}$.

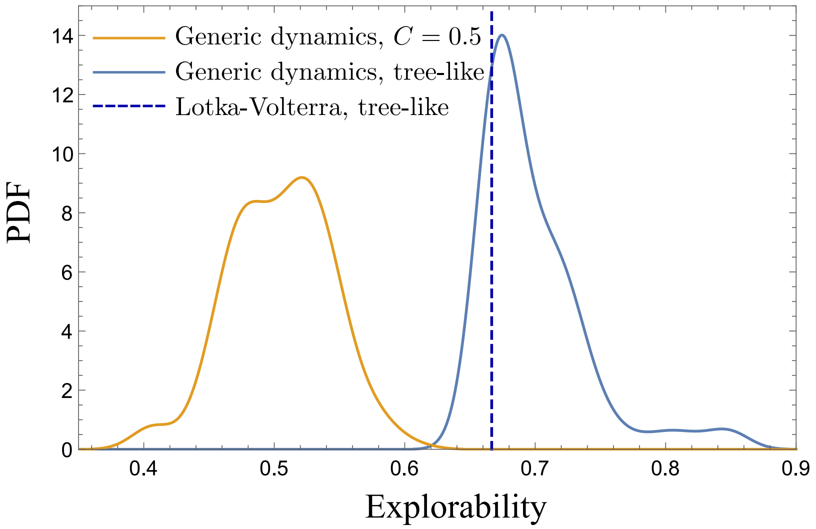

Figure S6: Probability distribution functions (PDF) of the explorability V_E for a generic form of the dynamics, Eq. (S8), for the optimal tree-like network (blue solid curve) and $C = 0.5$ (orange curve). Dashed-blue line indicates the explorability of the optimal tree-like network in the case of the Lotka-Volterra dynamics for comparison. Still for such a generic form of the dynamics, more dense networks customarily lead to lower values of the explorability. We performed 10^2 independent realizations of the added links ($\epsilon_{ij} = 0$ for the tree-like network), and for each one 10 different dynamics encoded in $\boldsymbol{\xi}$, extracted from a uniform distribution in $[0,1]^S$. Parameters are set to $S = 20$, $\alpha = 1$, $\sigma_\epsilon = 0.12$ and $\sigma_x = \sigma_\alpha = 0$.

3 Dynamical robustness

In this section we derive the explicit expression of the perturbed dynamics Jacobian (see Main Text). In addition, we discuss how to include additional information on the dynamics rather than performing a simple random-matrix approach, and include several numerical simulations to support this method.

3.1 Perturbing the dynamics

We introduce a perturbation of the dynamics, Eq. (S8), in the form $\dot{x}_i = (G_i + \delta G_i)(F_i + \delta F_i)(z_i)$ where $z_i = \sum_j w_{ij} x_j$. Up to the first order in the perturbation the dynamical equation becomes:

$$\dot{x}_i = G_i(x_i) F_i(z_i) + \delta G_i(x_i) F_i(z_i) + G_i(x_i) \delta F_i(z_i). \tag{S9}$$

The new fixed point equation becomes $(F_i + \delta F_i)(z_i'^*) = 0$. Now we suppose that the new fixed point $x_i^{*\prime}$ of (S9) differs only by a little amount δ_i from the original fixed point of the unperturbed dynamics, i.e. $x_i^{*\prime} = x_i^* + \delta_i$. In this case $z_i^{*\prime} \equiv \sum_j w_{ij} x_i^{*\prime} = z_i^* + \Delta_i$, with $\Delta_i \equiv \sum_j w_{ij} \delta_j$. Thus to the first order in δF we get

$$\delta_i = -\sum_j w_{ij}^{-1} \left. \frac{\delta F_j(z_j)}{dF_j(z_j)/dz_j} \right|_{z_j = z_j^*} \tag{S10}$$

We are now interested in calculating the Jacobian matrix evaluated at the fixed point. Reminding that, by definition, $F_i(z_i) = 0$ at $\mathbf{x}^*$, and that $\frac{dF_i(z_i)}{dx_j} = \frac{dF_i(z_i)}{dz_i}\frac{dz_i}{dx_j} = \frac{dF_i(z_i)}{dz_i} w_{ij}$. As $\frac{dG_i(x_i)}{dx_j} = \frac{dG_i(x_i)}{dx_i}\delta_{ij}$ and $\frac{d\delta G_i(x_i)}{dx_j} = \frac{d\delta G_i(x_i)}{dx_i}\delta_{ij}$, the Jacobian matrix evaluated at the new fixed point can be written as:

$$J'_{ij} = \xi'_i w_{ij}, \tag{S11}$$

where

$$\xi'_i = \left[G_i(x_i) \left(\frac{dF_i(z_i)}{dz_i} + \frac{d\delta F_i(z_i)}{dz_i} + \frac{d^2 F_i(z_i)}{d{z_i}^2} \Delta_i \right) + \frac{dF_i(z_i)}{dz_i} \left(\frac{dG_i(x_i)}{dx_i} \delta_i + \delta G_i(x_i) \right) \right]. \tag{S12}$$

Adopting a random matrix approach (i.e. without further information about the dynamics), ξ'_i can be set to be a random variable, that, in analogy with a Lotka-Volterra dynamics, we take uniformly distributed between 0 and 1 (see Main Text).

3.2 Including additional information in the perturbed Jacobian matrix

For the Lotka-Volterra dynamics, $\boldsymbol{\xi} = \mathbf{x}^*$. In general, we can suppose that there exists a correlation between the vectors $\boldsymbol{\xi}$ and its perturbed version $\boldsymbol{\xi}'$ with the fixed point $\mathbf{x}^*$. A

simple way to introduce such a correlation is:

$$\xi_i = \gamma r_i + (1-\gamma)x_i^* \tag{S13}$$
$$\xi_i' = \gamma' r_i' + (1-\gamma')x_i^* \tag{S14}$$

where r_i and r_i' are random variables uniformly distributed between 0 and 1, and γ and γ' are parameters (between 0 and 1) controlling the correlation for the non-perturbed and the perturbed dynamics, respectively.

Fig. S7 illustrates numerical results for the explorability when the unperturbed and the perturbed dynamics are respectively characterized by $\gamma = 0.1$ and $\gamma' = 0.5$ (with this choice, the unperturbed dynamics is "closer" to a Lotka-Volterra dynamics than the perturbed one). Analogously in Fig. S8 we show the probability distribution of the maximum real part of the eigenvalue of the Jacobian for the perturbed dynamics for the same choice of parameters. As it can be seen, our conclusions are still valid in this more general situation: tree-like networks generally offer the best performance. Qualitatively similar results are obtained for other values of γ and γ'.

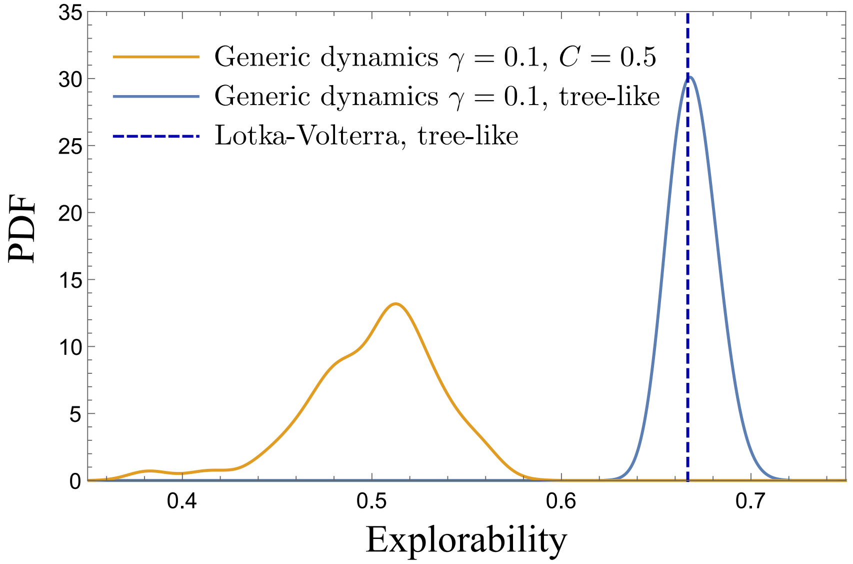

Figure S7: Probability distribution functions (PDF) of the explorability V_E for generic dynamics characterized by $\gamma = 0.1$ (Eq. (S13)). Solid blue and orange curves represent the optimal tree-like network and $C = 0.5$, respectively. Dashed blue line indicates the value of V_E for the optimal tree-like network in the case $\gamma = 0$. We performed 10^2 independent realizations of the added links ($\epsilon_{ij} = 0$ for the tree-like network), and for each one 10 different dynamics $\boldsymbol{\xi}$ as given by Eq. (S13). Parameters are set to $S = 20$, $\alpha = 1$, $\sigma_\epsilon = 0.12$ and $\sigma_x = \sigma_\alpha = 0$.

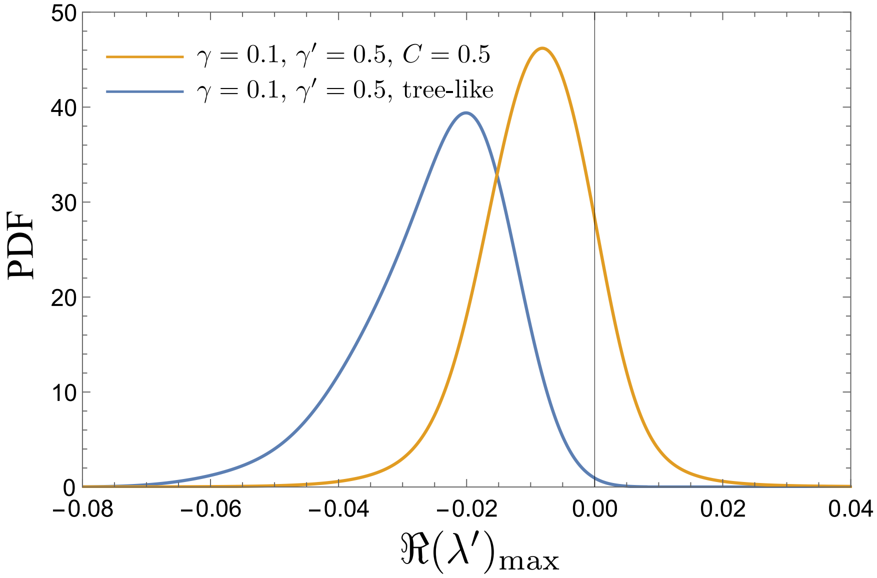

Figure S8: Probability distribution functions (PDF) of the real part of the maximum eigenvalue of the Jacobian matrix of the perturbed dynamics with $\gamma' = 0.5$ whereas the non-perturbed dynamics is characterized by $\gamma = 0.1$ (see Equations (S13) and (S14)). We performed 10^2 independent realizations of the added links ($\epsilon_{ij} = 0$ for the tree-like network), and for each one 10 different dynamics $\boldsymbol{\xi}$. For each matrix at the edge of instability (within an error $|\Re(\lambda')_{\max}| < 10^{-2}$), we sampled over 10^3 perturbed dynamics $\boldsymbol{\xi'}$. Parameters are set to $S = 20$, $\alpha = 1$, $\sigma_\epsilon = 0.12$ and $\sigma_x = \sigma_\alpha = 0$.

3.3 Measuring dynamical robustness

When analyzing the dynamical robustness, we qualitatively compare the PDF of $\Re(\lambda')_{\max}$ for different connectivities. Based on such distribution, we can give a quantitative measure R of "how dynamically robust" is a certain topology, that will be useful when implementing the optimization algorithm.

In the Main text we refer to the 5th percentile (with a minus sign) of the distribution of $\Re(\lambda')_{\max}$ as a measure of dynamical robustness, $R = -\Re(\lambda')^{5th}_{\max}$ (with the minus sign, more robust topologies lead to larger values of R). Naively speaking, this is a quantification of "how stable" can be the system for such perturbed dynamics that makes the system as stable as possible. Certainly, it is possible to define other measures of this property (e.g. the 10th, 20th and 50th percentiles) that lead, qualitatively, to the same conclusion, as illustrated in Fig. S9. However, lower percentile values generally enhance the differences between topologies.

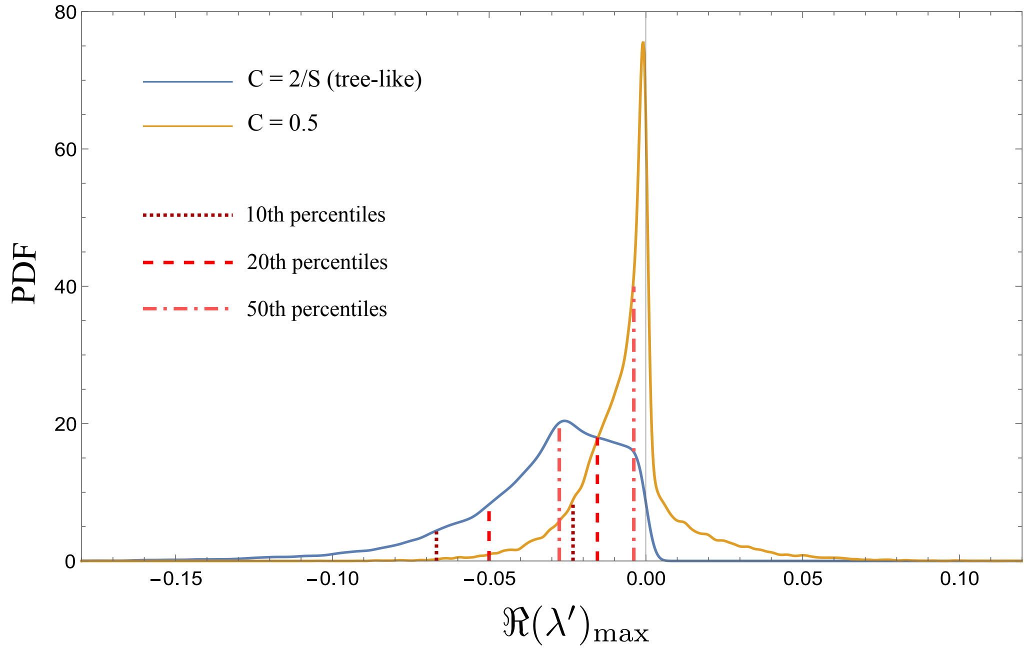

Figure S9: Comparison between different measures of dynamical robustness (10th, 20th and 50th percentiles of the distribution $P(\Re(\lambda')_{\max})$) in the case of $\sigma_\alpha = \sigma_x = 0.1$ for the optimal tree-like network and graphs of $S = 20$ with $C = 0.5$ (for which $\sigma_\epsilon = 0.12$). We have set $\alpha = 1$. All the curves are obtained as explained in Fig. 3 of the main text. In all cases, the corresponding measure of dynamical robustness decreases when increasing the connectivity.

4 From sparse interaction matrices to community structures

An important characteristic of sparse tree-like is their ability to remain sparse after aggregation of many of them. Therefore, sparse systems will preserve their optimal features after aggregation. More specifically, the characteristic polynomial only depends on the loops of the network (see Section 2.1), and therefore an aggregation of sparse structures through a few links will not dramatically change its explorability. In Fig. S10 we represent a simple example of two tree-like topologies that can be assembled, preserving the optimality feature.

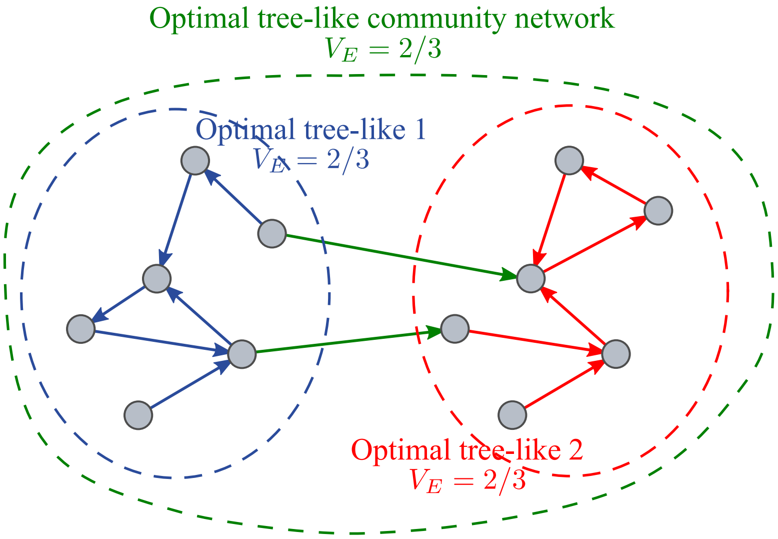

Figure S10: Example of two tree-like networks that can be assembled obtaining a larger community with the same explorability as each single component.

References

[1] GJ Ackland and ID Gallagher. Stabilization of large generalized lotka-volterra foodwebs by evolutionary feedback. *Physical review letters*, 93(15):158701, 2004.

[2] Hisao P Arai and Dwight R Mudry. Protozoan and metazoan parasites of fishes from the headwaters of the parsnip and mcgregor rivers, british columbia: a study of possible parasite transfaunations. *Canadian Journal of Fisheries and Aquatic Sciences*, 40(10):1676–1684, 1983.

[3] Ralph V Bangham. Studies on fish parasites of lake huron and manitoulin island. *American Midland Naturalist*, pages 184–194, 1955.

[4] FA Berezin. *The method of second quantization*, volume 24. Elsevier, 2012.

[5] Immanuel M Bomze. Lotka-volterra equation and replicator dynamics: a two-dimensional classification. *Biological cybernetics*, 48(3):201–211, 1983.

[6] Vanni Bucci and Joao B Xavier. Towards predictive models of the human gut microbiome. *Journal of molecular biology*, 426(23):3907–3916, 2014.

[7] VC Chinniah and William Threlfall. Metazoan arasites of fish from the smallwood reservoir, labrador, canada. *Journal of Fish Biology*, 13(2):203–213, 1978.

[8] Katharine Z Coyte, Jonas Schluter, and Kevin R Foster. The ecology of the microbiome: Networks, competition, and stability. *Science*, 350(6261):663–666, 2015.

[9] Alex O Dechtiar. Parasites of fish from lake of the woods, ontario. *Journal of the Fisheries Board of Canada*, 29(3):275–283, 1972.

[10] Masayuki Hirafuji, Kei Tanaka, and Scott Hagan. Lotka-volterra machine for a general model of complex biological systems. In *Computer Aided Control System Design, 1999. Proceedings of the 1999 IEEE International Symposium on*, pages 516–521. IEEE, 1999.

[11] Anthony Joern. Feeding patterns in grasshoppers (orthoptera: Acrididae): factors influencing diet specialization. *Oecologia*, 38(3):325–347, 1979.

[12] Simon R Leather. Feeding specialisation and host distribution of british and finnish prunus feeding macrolepidoptera. *Oikos*, pages 40–48, 1991.

[13] TS Leong and JC Holmest. Communities of metazoan parasites in open water fishes of cold lake, alberta. *Journal of Fish Biology*, 18(6):693–713, 1981.

[14] Yang-Yu Liu, Jean-Jacques Slotine, and Albert-László Barabási. Controllability of complex networks. *Nature*, 473(7346):167–173, 2011.

[15] Robert M May. Will a large complex system be stable? *Nature*, 238:413–414, 1972.

[16] Jose C Nacher and Tatsuya Akutsu. Structural controllability of unidirectional bipartite networks. *Scientific reports*, 3, 2013.

[17] Alan Roberts. The stability of a feasible random ecosystem. *Nature*, 251:607–608, 1974.

[18] Lewi Stone. The google matrix controls the stability of structured ecological and biological networks. *Nature Communications*, 7, 2016.

[19] Samir Suweis, Filippo Simini, Jayanth R Banavar, and Amos Maritan. Emergence of structural and dynamical properties of ecological mutualistic networks. *Nature*, 500(7463):449–452, 2013.

[20] Frank L Tobin, Valeriu Damian-Iordache, and Larry D Greller. Towards the reconstruction of gene regulatory networks. In *Technical Proc. 1999 International Conference on Modeling and Simulation of Microsystems*. Citeseer, 1999.
